# GAA‐*FGF14* Ataxia Is a Frequently Overlooked Cause of Sporadic Adult‐Onset Ataxia

**DOI:** 10.1111/cge.70184

**Published:** 2026-05-28

**Authors:** Eva‐Maria Kraus, Johannes Lenz, Pauline Ploettner, Patricia Duffek, Jost‐Julian Rumpf, Rami Abou Jamra, John Wiedenhoeft, Denny Popp

**Affiliations:** ^1^ Institute of Human Genetics University of Leipzig Medical Center Leipzig Germany; ^2^ Department of Neurology University of Leipzig Medical Center Leipzig Germany

**Keywords:** ataxia, GAA‐*FGF14*‐ataxia, nanopore sequencing, repeat expansion disorders, SCA27B, spinocerebellar ataxia 27B

## Abstract

GAA‐*FGF14* ataxia (spinocerebellar ataxia 27B, SCA27B), identified in 2023, is a major cause of adult‐onset autosomal dominant cerebellar ataxia (ADCA). In this study, we assessed the frequency of GAA‐*FGF14* ataxia in a predominantly sporadic German cohort of 107 genetically unresolved index patients using long‐range PCR and nanopore sequencing. Somatic mosaicism of GAA repeat length was assessed using a custom bioinformatics pipeline based on a modified cyclic Smith–Waterman algorithm. This approach provided streamlined detection and enabled precise genotyping. Among sporadic cases, 10% had a pathogenic and 6% an intermediate repeat expansion. Across the entire cohort, 13% carried a pathogenic and 5% an intermediate repeat expansion. Diagnostic yield varied substantially by clinical presentation: 50% in cases with typical GAA‐*FGF14*‐ataxia features, 13% in patients with compatible but less characteristic features and 5% in atypical presentations. In conclusion, this study further corroborates existing evidence that GAA‐*FGF14* ataxia is a frequent cause of both sporadic and familial cerebellar ataxia. Given its high diagnostic yield and the limited detectability by standard short‐read genome sequencing, targeted testing should be more widely implemented.

## Graphical abstract

GAA‐*FGF14* ataxia is a frequent cause of both familial and sporadic cerebellar ataxia. If symptoms are consistent, targeted testing of the *FGF14* locus should be considered as a first‐line approach, as the diagnostic yield is up to 50%.

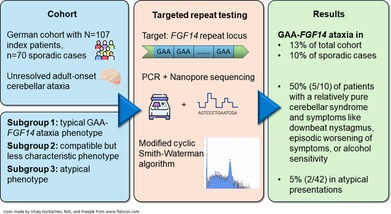

## 1 Introduction

GAA‐*FGF14* ataxia (spinocerebellar ataxia 27B, SCA27B, OMIM #620174) is caused by intronic GAA repeat expansions in the gene *FGF14*. Since its discovery in 2023, it has emerged as a major cause of adult‐onset autosomal dominant cerebellar ataxia (ADCA) in the European population. In two German cohorts, pathogenic *FGF14* expansions were identified in 23%–31% of ADCA cases [1, 2]. Moreover, pathogenic *FGF14* expansions were found in sporadic late‐onset cerebellar ataxia (SLOCA) cohorts from France (12.7%) [3], Greece (8.7%) [4], and Germany (11.9%) [5]. Clinically, it typically presents as a relatively pure cerebellar syndrome, often accompanied by downbeat nystagmus and episodic symptoms. Many patients report symptomatic benefit from 4‐aminopyridine [6, 7, 8].

As observed in other repeat expansion disorders, the GAA repeat expansion in *FGF14* was recently found to often occur as somatic mosaicism in terms of a spectrum of repeat lengths [9, 10]. The presence of such a spectrum complicates genotyping in diagnostic settings where distinct thresholds for a single (possibly) pathogenic repeat length are defined, hence necessitating a more refined calling strategy.

Here, we assessed the frequency of GAA‐*FGF14* ataxia in a predominantly sporadic German cohort using targeted long‐read sequencing and a customized bioinformatics pipeline, which revealed the spectrum of somatic mosaicism of GAA repeat length and enabled accurate genotyping.

## 2 Methods

### 2.1 Cohort and Groups Definition

We enrolled genetically unsolved cerebellar ataxia (CA) cases sent to our Institute of Human Genetics between November 2016 and September 2024. Inclusion criteria were progressive and/or episodic CA, an age at onset (AAO) > 18 years, and patients' written informed consent for further genetic analyses. Exclusion criteria were the identification of another pathogenic variant explaining the phenotype or a confirmed non‐genetic (acquired) cause convincingly explaining the symptoms. For details on cohort composition see Supporting Information: Text S1 (including Figures S1–S4).

All cases had previously undergone panel, exome, or genome sequencing. Additionally, all were tested for common repeat expansion disorders, including at least SCA1, SCA2, SCA3, and SCA6 (see Table S1 for details), except for six cases with episodic ataxia. Neurological evaluation was performed prior to genetic testing. Clinical data and family history were mainly extracted from medical reports.

Patients were categorized into three subgroups based on clinical presentation are as follows:

1. Typical GAA‐*FGF14* ataxia phenotype: A relatively pure cerebellar syndrome with an AAO > 30, slow progression and at least one of the following: downbeat nystagmus, episodic worsening of symptoms, alcohol sensitivity.
2. Matching but less characteristic phenotype: A relatively pure cerebellar syndrome with an AAO > 30 and slow progression, in the absence of one or more of the following: downbeat nystagmus, episodic worsening, or alcohol sensitivity (as far as reported).
3. Atypical phenotype: Disease progression inconsistent with GAA‐*FGF14* ataxia, for example, early‐onset, rapid progression, predominant extracerebellar features.

### 2.2 Genetic Screening for GAA‐*FGF14* Ataxia

We performed targeted sequencing of the *FGF14* repeat locus using DNA isolated from EDTA blood samples. Parts of intron 1 of the *FGF14* gene were amplified by PCR and sequenced using Nanopore sequencing (Oxford Nanopore Technologies). The reads were aligned against the human reference genome build hg38. For data analysis, we used the Integrative Genomics Viewer (IGV) and a customized bioinformatics pipeline using a cyclic variant of the Smith–Waterman algorithm [11]. The local modal values of the repeat sizes were used for clinical interpretation. A detailed description of the bioinformatic pipeline, outlining its specific strengths and limitations, is provided in (Text S1 and Table S2). Expansions > 250 GAA repeats were considered pathogenic, with 250–299 repeats associated with reduced penetrance [12]. Repeat counts between 180 and 249 were regarded as intermediate expansions and evaluated as variants of uncertain significance (VUS) [2].

## 3 Results

### 3.1 Total Cohort and Frequency of GAA‐*FGF14* Ataxia

In total, we enrolled 107 index patients with genetically undetermined adult‐onset CA. Only 15% (16/107) reported affected first‐degree relatives, whereas in 65% (70/107) family history was negative and in 20% (21/107) uninformative or unknown. Median AAO was 55 years (range 19–77 years).

**FIGURE 1:**
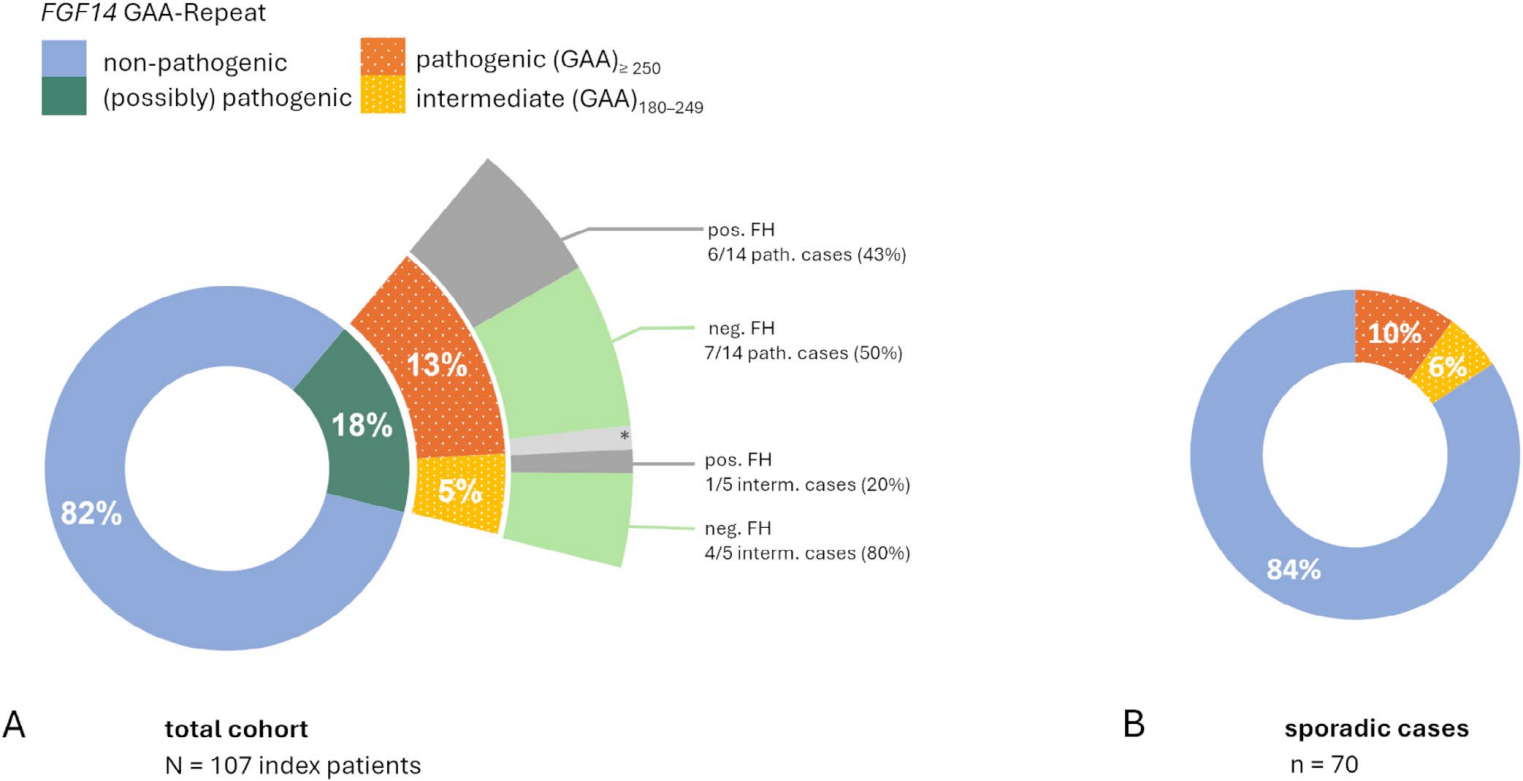
(A) Frequency of GAA‐*FGF14* ataxia in the total cohort distributed to pathogenic and intermediate cases (middle ring) and by family history (outer ring). *Family history unknown (1/14 pathogenic cases) (B) Frequency of GAA‐*FGF14* ataxia among sporadic cases.

A pathogenic *FGF14* expansion was found in 13% (14/107) of all cases. Of those, median AAO was 65 years (range 40–74 years). Expansion length ranged from 262 to 469 GAA repeats with three cases between 250 and 300 (reduced penetrance) and 11 cases with more than 300 repeats. In 43% of the pathogenic cases family history was positive, in 50% negative and in one case unknown. Intermediate repeat expansions were found in 5% (5/107) of the cases, ranging from 183 to 213 repeats (Figure 1A).

In two of the intermediate cases, an additional VUS was identified. Long‐read genome sequencing revealed an intermediate *CNBP* repeat expansion in ID37, possibly explaining the patients' additional muscular phenotype. In addition, short‐read genome sequencing revealed an uncertain variant in the *ELOVL5* gene in ID35, which may, if clinically relevant, contribute to the ataxia (for details see Table S3).

### 3.2 Diagnostic Yield in Clinical Subgroups

**FIGURE 2:**
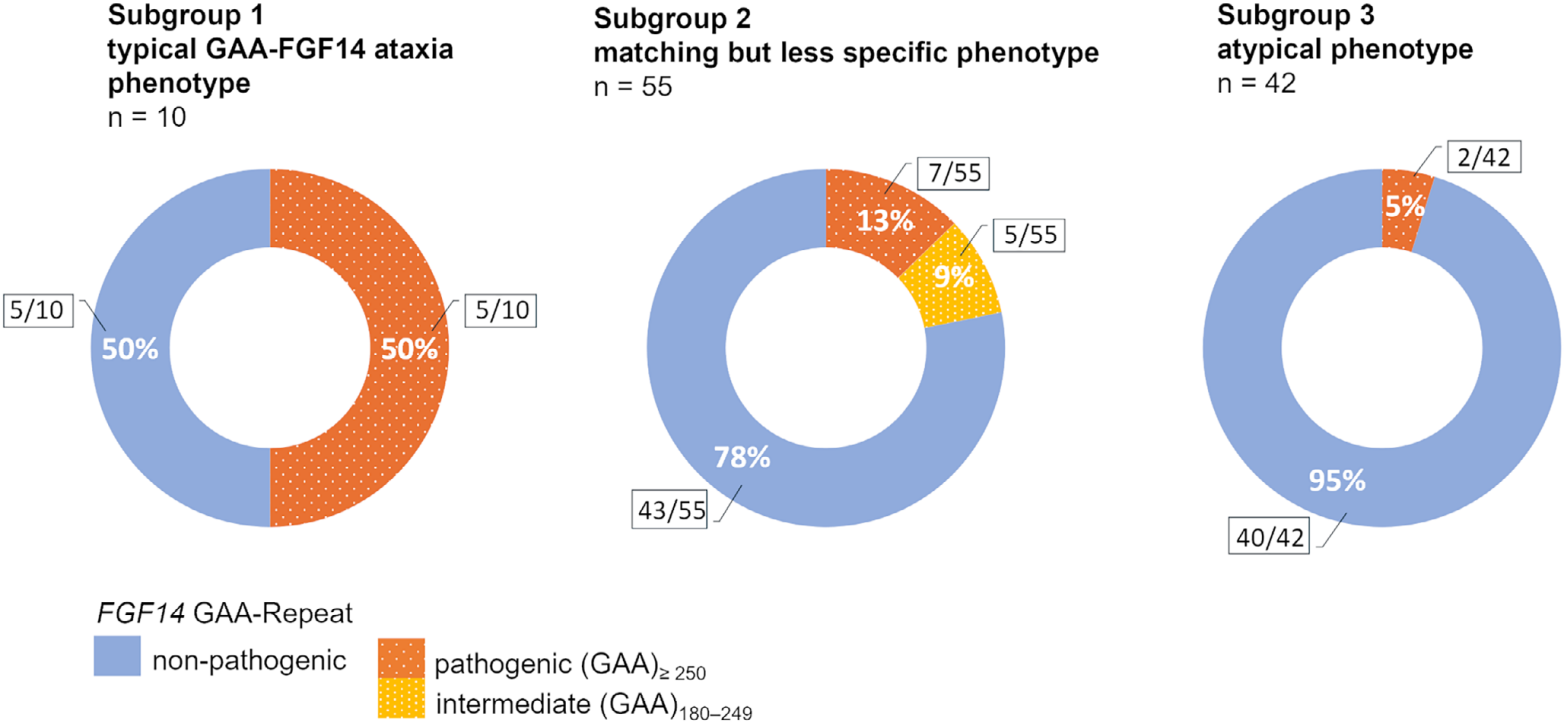
GAA‐*FGF14* ataxia in symptom‐based patient groups. Subgroup 1: CA + downbeat nystagmus/episodic component/alcohol sensitivity. Subgroup 2: Relatively pure CA with AAO > 30 years. Subgroup 3: CA but rather atypical symptoms for GAA‐*FGF14* (e.g., rapid progression, prominent extracerebellar symptoms).

Diagnostic yield varied clearly by clinical presentation. Among patients with at least one typical GAA‐*FGF14* ataxia feature (Subgroup 1), a pathogenic GAA‐*FGF14* expansion was found in 50%. Among patients with a relatively pure cerebellar ataxia but without reported characteristic features such as downbeat nystagmus or an episodic component (Subgroup 2), 13% (7/55) carried a pathogenic and 9% (5/55) an intermediate expansion. Among patients with a rather atypical phenotype (Subgroup 3), 5% (2/42) had a pathogenic expansion. A fully penetrant expansion was identified in a patient with diagnosed atypical parkinsonism with a prominent hypokinetic‐rigid syndrome and a mild cerebellar syndrome (ID69). In addition, a pathogenic expansion with reduced penetrance was found in a patient with alternating episodic symptoms including pain, paresthesia, weakness, and vertigo since the age of 40 (ID104) (Figure 2 and Table S3).

### 3.3 GAA‐*FGF14* Ataxia Among Sporadic Cases

Among the sporadic unsolved ataxia cases in total, 10% (7/70) carried a pathogenic and additionally 6% (4/70) an intermediate repeat expansion in *FGF14* (Figure 1B).

Stratified by clinical subgroups, GAA‐*FGF14* ataxia was identified in 33% (2/6) of sporadic cases in Subgroup 1 and in 8% (3/38) of sporadic cases in Subgroup 2. Additionally, 10% (4/38) of Subgroup 2 carried an intermediate expansion. In Subgroup 3, both GAA‐*FGF14* cases were sporadic. A visual stratification of sporadic cases by clinical subgroups is provided in Figure S5.

## 4 Discussion

We identified GAA‐*FGF14* ataxia in 13% of unresolved adult‐onset ataxia cases sent to the Institute of Human Genetics in Leipzig. Notably, in < 50% of the pathogenic cases, a positive family history was reported. These findings further support existing evidence that pathogenic GAA‐*FGF14* expansions represent one of the most frequent genetic causes of adult‐onset ataxia, even in the absence of an autosomal‐dominant inheritance pattern. Moreover, the SCA27B detection rate and the clinical description in our study substantially match those of previously reported papers [3, 4, 5].

It is important to note that clinical features and family history were primarily obtained from routine clinical documentation (e.g., medical reports and laboratory requests) and therefore could not be collected in a standardized manner. Consequently, some clinical features may have been overlooked during prior (nontargeted) neurological examinations. This may especially limit the interpretability of the findings regarding the diagnostic yield in different symptom‐based subgroups. Moreover, cases classified as sporadic may, in fact, represent undetected familial cases. However, these limitations reflect real‐world clinical circumstances and, therefore, offer pragmatic, practice‐oriented insights into the diagnostic yield. Moreover, several biological factors may obscure inheritance patterns, including late disease onset, slow progression, and intergenerational instability of GAA‐*FGF14* repeat expansions. The latter typically involves expansion during maternal and contraction during paternal transmission [13, 14], often resulting in an apparently sporadic presentation. Finally, it should be noted that this study is restricted to a cohort of adult‐onset ataxia patients who remained genetically undiagnosed after standard diagnostic testing. This limits the extrapolation to unselected ataxia cohorts or to earlier stages of the diagnostic pathway.

Given the high diagnostic yield, targeted testing of the *FGF14* locus should be considered a first‐line approach in patients with typical clinical presentations and typical features like downbeat nystagmus. This strategy offers a faster and more cost‐effective diagnostic pathway compared to untargeted approaches like whole genome sequencing (WGS). Moreover, accumulating evidence shows that *FGF14* repeat expansions are not detectable by short‐read sequencing technologies, on which standard WGS relies [13, 15, 16], and most laboratories have not included *FGF14* in their repeat‐expansion panels yet. For this reason, and especially given the availability of a promising symptomatic treatment option, the broad implementation of an easily accessible diagnostic test for GAA‐*FGF14* ataxia is of particular clinical importance. A discussion of our analytical strategy, including a brief contextualization with alternative sequencing methods, is provided in the Supporting Information.

The two atypical cases in our series merit further clinical evaluation. While the *FGF14*‐expansion may play a contributory role, additional genetic, or environmental factors are probably required to explain the full phenotype.

Regarding the five cases harboring intermediate GAA‐*FGF14* expansions, it is important to note that the clinical significance of intermediate alleles remains uncertain [2, 8, 17]. Although all patients presented with a slowly progressive, relatively pure cerebellar syndrome, the individual clinical manifestations were heterogeneous. This also applies to the three cases with biallelic expansions (ID22, ID45, and ID57). Although some biallelic pathogenic GAA‐*FGF14* cases showed earlier onset and faster progression [6, 14, 18], our cases involve only intermediate expansions with currently unclear clinical relevance. Consequently, no meaningful conclusions can be drawn at this stage. Accordingly, this group requires longitudinal neurological surveillance, including ongoing assessment for the GAA‐*FGF14* phenotype. In the two cases with an additional VUS in another gene, the associated phenotype should be carefully evaluated. From a genetic perspective, all variants classified as uncertain should be re‐evaluated every 12–24 months. Predictive testing of asymptomatic relatives for VUS is generally not recommended. This aspect should be addressed within the framework of genetic counseling. Finally, in patients with intermediate GAA‐*FGF14* expansions, we recommend comprehensive genetic testing for other hereditary ataxias to achieve diagnostic certainty.

## 5 Conclusion

Our findings further support the role of GAA‐*FGF14* expansions as a frequent, yet underdiagnosed, cause of cerebellar ataxia. Implementing early genetic screening is essential for accurate diagnosis and informed therapeutic management in routine clinical practice.

## Author Contributions

E.K.: conceptualization, implementation, data acquisition, cohort formation, analysis, drafting, and revision. J.L.: analysis, critical revision. P.P. and J‐.J.R.: clinical data acquisition, critical revision. P.D.: molecular analysis, critical revision. R.A.J.: conceptualization, critical revision. J.W.: bioinformatics, drafting, and revision. D.P.: conceptualization, implementation, analysis, drafting, and revision.

## Funding

The authors have nothing to report.

## Ethics Statement

We confirm that this work complies with the ethics and integrity policies of the journal. Written informed consent was obtained by the clinician or human geneticist from all patients and documented in the medical records. The study was conducted in accordance with the Declaration of Helsinki and approved by the Ethics committee of the University of Leipzig Medical Center as part of the study “Genetics of rare diseases based on Next Generation Sequencing” (approval number 402/16‐ek, July 9, 2021). Individual patients cannot be identified from the genetic or clinical data presented.

## Conflicts of Interest

The authors declare no conflicts of interest.

## Supporting information


**FIGURE S1:** Gel electrophoresis of PCR products. GAA repeat numbers per allele were determined by the modified cyclic Smith–Waterman algorithm.
**FIGURE S2:** Exemplary tandem repeat spectrum of the GAA repeats in *FGF14*.
**FIGURE S3:** Number of repeats in relation to the number of repeats in the overhang.
**FIGURE S4:** Number of repeats in the reverse‐complement reads in relation to the number of repeats in the original read.
**FIGURE S5:** GAA‐*FGF14* ataxia among sporadic cases stratified by clinical subgroups.
**TEXT S1:** Extended Methods: Details about cohort formation, DNA sequencing, application of the CSW, and the resulting tandem repeat spectrum (including Figures S1–S4).
**TEXT S2:** Discussion of the analytical strategy: Alternative sequencing methods and performance of the CSW algorithm.
**TEXT S3:** GAA‐*FGF14* ataxia among sporadic cases stratified by clinical subgroups (including Figure S5).

**TABLE S1:** Overview of prior genetic testing for all patients.
**TABLE S2:** Examples of exact genotyping using IGV and CSW.
**TABLE S3:** Clinical and genetic details of the GAA‐*FGF14* ataxia cases.

## Data Availability

The data that support the findings of this study are available from the corresponding author upon reasonable request.
