# Supplementary material for: GAA‐FGF14 Ataxia Is a Frequently Overlooked Cause of Sporadic Adult‐Onset Ataxia
Source: Clin Genet. 2026 May 28;110(3):358–62. doi: 10.1111/cge.70184 (PMC13432038; doi:10.1111/cge.70184)
Supplement: Supplementary file 1 — FIGURE S1: Gel electrophoresis of PCR products. GAA repeat numbers per allele were determined by the modified cyclic Smith–Waterman algorithm. FIGURE S2: Exemplary tandem repeat spectrum of the GAA repeats in FGF14. FIGURE S3: Number of repeats in relation to the number of repeats in the overhang. FIGURE S4: Number of repeats in the reverse‐complement reads in relation to the number of repeats in the original read. FIGURE S5: GAA‐FGF14 ataxia among sporadic cases stratified by clinical subgroups. TEXT S1: Extended Methods: Details about cohort formation, DNA sequencing, application of the CSW, and the resulting tandem repeat spectrum (including Figures S1–S4). TEXT S2: Discussion of the analytical strategy: Alternative sequencing methods and performance of the CSW algorithm. TEXT S3: GAA‐FGF14 ataxia among sporadic cases stratified by clinical subgroups (including Figure S5). [file CGE-110-358-s001.docx]

Supplementary material to

GAA-*FGF14* ataxia is a frequently overlooked cause of sporadic adult‐onset ataxia

Eva-Maria Kraus, MD^1^, Johannes Lenz^1^, Pauline Ploettner, MD^2^, Patricia Duffek^1^, Jost-Julian Rumpf, MD^2^, Rami Abou Jamra, MD^1^, John Wiedenhoeft^*^, PhD^1^, Denny Popp^*^, PhD^1^

^1^Institute of Human Genetics, University of Leipzig Medical Center, Leipzig, Germany.

^2^Department of Neurology, University of Leipzig Medical Center, Leipzig, Germany.

*D.P. and J.W. should be considered joint senior author.

Corresponding authors: Eva-Maria Kraus or Denny Popp, Institute of Human Genetics, University of Leipzig Medical Center, Philipp-Rosenthal-Straße 55, 04103 Leipzig, Germany. E-Mail: eva-maria.kraus@medizin.uni-leipzig.de, denny.popp@medizin.uni-leipzig.de

Supplementary material

Extended methods

Detailed cohort formation process

We filtered our in-house patient data base for unsolved cases that exhibited at least one of the following Human Phenotype Ontology (HPO) terms: *ataxia* (HP:0001251), *abnormality of coordination* (HP:0011443), *episodic ataxia* (HP:0002131), *nystagmus* (HP:0000639), *downbeat nystagmus* (HP:0010545), *upbeat nystagmus* (HP:0011477), *late-onset spinocerebellar degeneration* (HP:0006904), *nonprogressive cerebellar ataxia* (HP:0002470), *truncal ataxia* (HP:0002078), *gait ataxia* (HP:0002066), *dysmetria* (HP:0001310), *dysdiadochokinesis* (HP:0002075), *limb ataxia* (HP:0002070), *gait disturbance* (HP:0001288), *vertigo* (HP:0002321), *tremor* (HP:0001337), *gait imbalance* (HP:0002141), or *progressive cerebellar ataxia* (HP:0002073). Cases were further limited to those classified under the in-house disease group categories “ataxia”, “neurodegenerative disease” or “unspecified movement disorder”. To reduce the risk of excluding relevant cases due to potential misclassification, cases lacking a defined disease group category were also included as well as those assigned to the disease group categories “parkinson”, “tremor” or “spastic paraplegia”. The resulting primary cohort was subsequently manually screened according to the study’s predefined inclusion and exclusion criteria (see Methods).

DNA extraction, amplification and Nanopore sequencing

Genomic DNA (gDNA) was extracted from EDTA whole blood using the MagCore® HF 16 Plus II Nucleic Acid Extractor. Starting from 400 µl EDTA blood samples 100 µl DNA elution volume was obtained using the Genomic DNA Whole Blood Kit (RBC Bioscience®) and 101 cartridge (RBC Bioscience®) according to the manufacturers’ instructions. DNA concentration and purity (260/280 ratio) were measured with a NanoDrop 2000 (Thermo Scientific) or a Tecan Infinite^®^ 200 Microplate Reader using the absorbance mode.

Part of the *FGF14* gene (NM_001321939.2) was amplified by PCR using primers binding in intron 1 (forward primer sequence: AGCAATCGTCAGTCAGTGTAAGC; reverse primer sequence: CAGTTCCTGCCCACATAGAGC as described in Mohren et al.^1^. Size of PCR products were checked by gel electrophoresis. A GAA repeat expansion in *FGF14* could be presumed by the gel electrophoresis in all cases, see Figure S1 for examples.


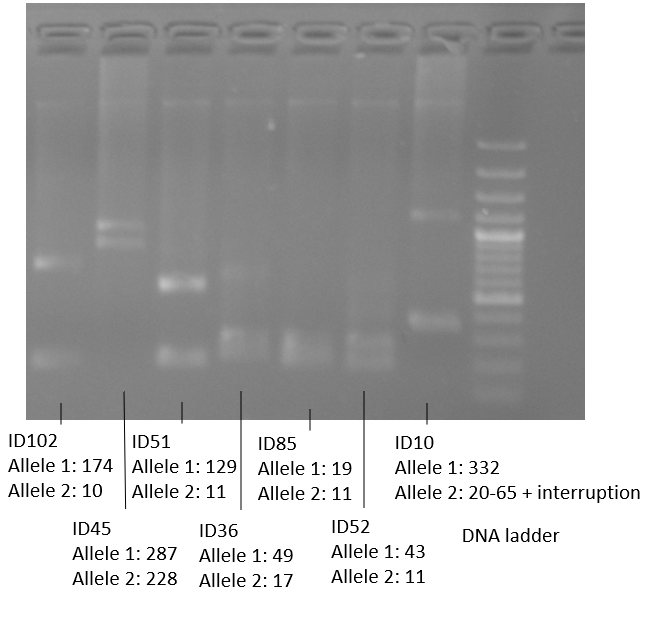


Figure S1: Gel electrophoresis of PCR products. GAA repeat numbers per allele were determined by the modified cyclic Smith-Waterman algorithm.

Libraries for long-read sequencing were prepared from the PCR products using the NEBNext Companion Module (New England Biolabs, Frankfurt am Main, Germany) and the 96 barcoding library prep kit SQK-NBD114.96 according to the standard protocol of Oxford Nanopore Technology (ONT, Oxford, UK) with the following adjustments. Incubation time of the end-prep reaction was increased to 15 min at 20 °C and 15 min at 65 °C. Incubation time of barcode ligation was adapted to 20 min. Incubation time of adapter ligation reaction was adjusted to 30 min at room temperature. Final libraries were loaded onto PromethIon flow cells of type R10.4.1 and sequenced on a P2Solo device (ONT). Raw data (fast5 files) were basecalled, de-multiplexed and aligned against the human reference genome hg38 using dorado 0.8.1 in super accuracy mode (ONT).

Exact genotyping using the IGV and an alternative cyclic Smith-Waterman algorithm

After targeted sequencing of the *FGF14* repeat locus and alignment of the reads to the human reference genome, all results were visualized in IGV to assess (i) approximate repeat length, (ii) repeat sequence and (iii) possible interruptions. IGV proved highly effective for initial screening, allowing us to distinguish between clearly negative and potentially positive cases. However, due to the known strong somatic mosaicism^1^, no single representative repeat length could be determined. Rather, it became clear that the repeat lengths often follow a wide, multimodal distribution. For a more accurate assessment, we implemented a cyclic version of the Smith-Waterman algorithm^2^ (CSW) for quantification of the repeat length within each read. Plotting these repeat lengths resulted in a tandem repeat spectrum which enabled the most accurate possible estimation of expansion size despite the underlying somatic mosaicism. However, all cases still required manual validation with IGV to confirm the GAA repeat motif and approximate repeat length. Local modal values were used for clinical interpretation (for details and visual examples see Table S2).

Cyclic Smith-Waterman algorithm (CSW)

The classic Smith-Waterman algorithm (SW) for local alignment^2^ (understood as its quadratic time and space variant due to Gotoh^3^, as usual), defines recursion relations that allow for the computation of local alignments in a single pass over a dynamic programming matrix DP. The classic book by Durbin et al.^4^ provides several generalizations to multiple matches, semi-global and overlapping alignments, as well as tandem repeats. We used a modified, cyclic version for finding a single local stretch of tandem repeats, which differs slightly from the published version due to concerns about its correctness in some edge cases. Our algorithm also includes a counting step which does not rely on any particular basis in the motif being considered its “first”.

Application of CSW

For each read, we used CSW to compute the longest tandem repeat on both the sequenced strand as well as its reverse-complement, and selected whichever one yielded a higher repeat count. This leads to a potential overestimation in very short repeat lengths in cases where the true repeat sequence is extremely short or too severely degraded to be detected while exhibiting sporadic repeats on the reverse strand. However, this bias is expected at repeat lengths well below the clinical threshold, and can be assessed visually (see below). Repeats which aligned with the start or end of the read were marked as potentially incomplete.

**
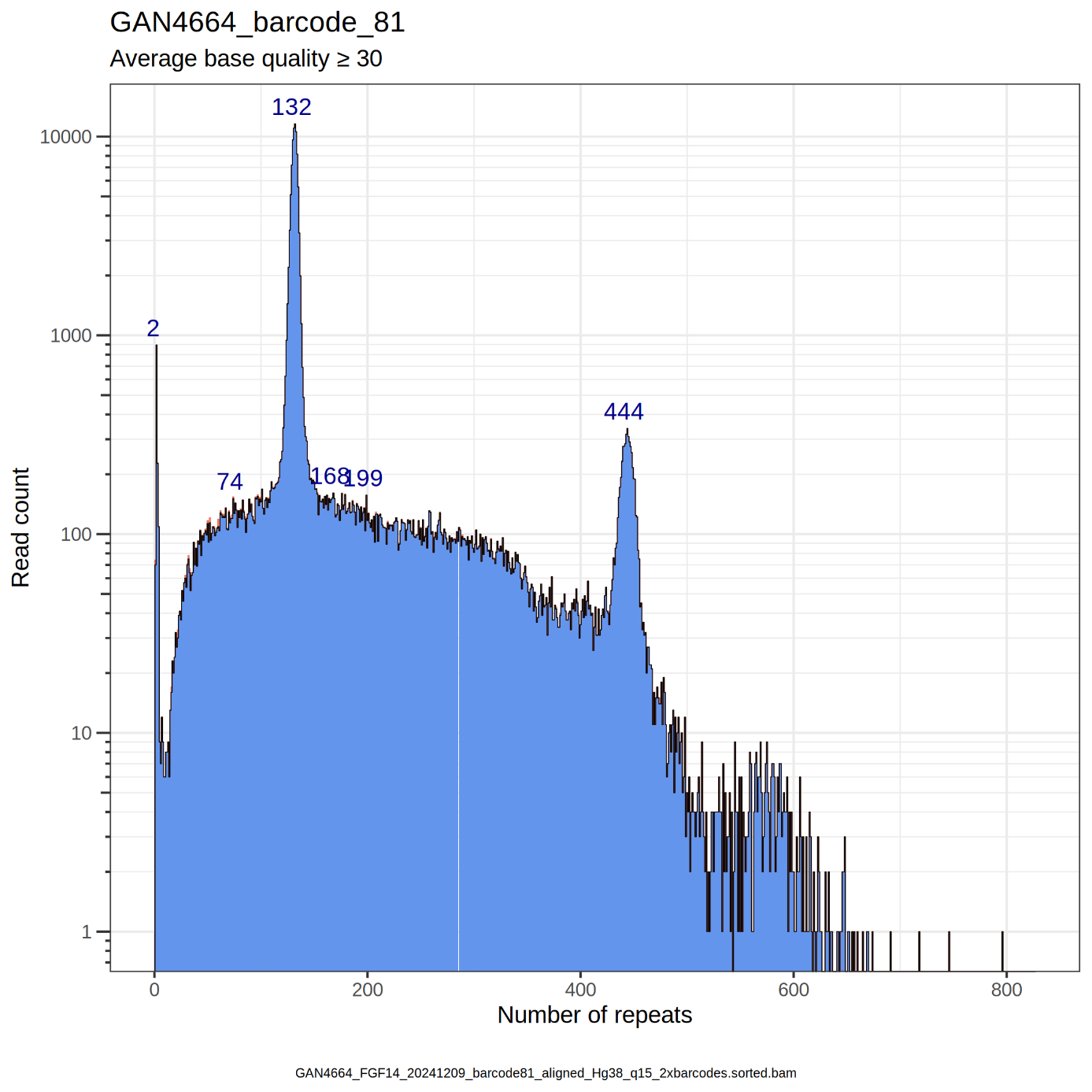
**

Figure S2: Exemplary tandem repeat spectrum of the GAA repeats in *FGF14*

Aggregating counts for each repeat length yields the *tandem repeat spectrum*, see Figure S2. The counts of all repeat lengths across all reads with an average PHRED score of at least 30 were plotted, with those of potentially incomplete reads marked in red on top of the spectrum. The counts were log10-transformed, as the generating process appears to follow some power-law distribution. We used a simple sliding-window approach for automated peak annotation. The local modal values of the repeat sizes were used for clinical interpretation.

Additional plots were created to assess the quality of the calls and safeguard against known failure modes of CSW: In cases where a stretch of tandem repeats is interrupted by a low-scoring region due to DNA insertion or degraded sequence signal, CSW might miss the shorter part of that stretch due to calling a local alignment. We assessed this by running CSW on the uncalled regions of the read (called overhangs) as well, and plotted the sizes of the longest tandem repeats on either side against the one being called. For high-quality calls, we expected to find few cases at or below the main diagonal, see Figure S3.


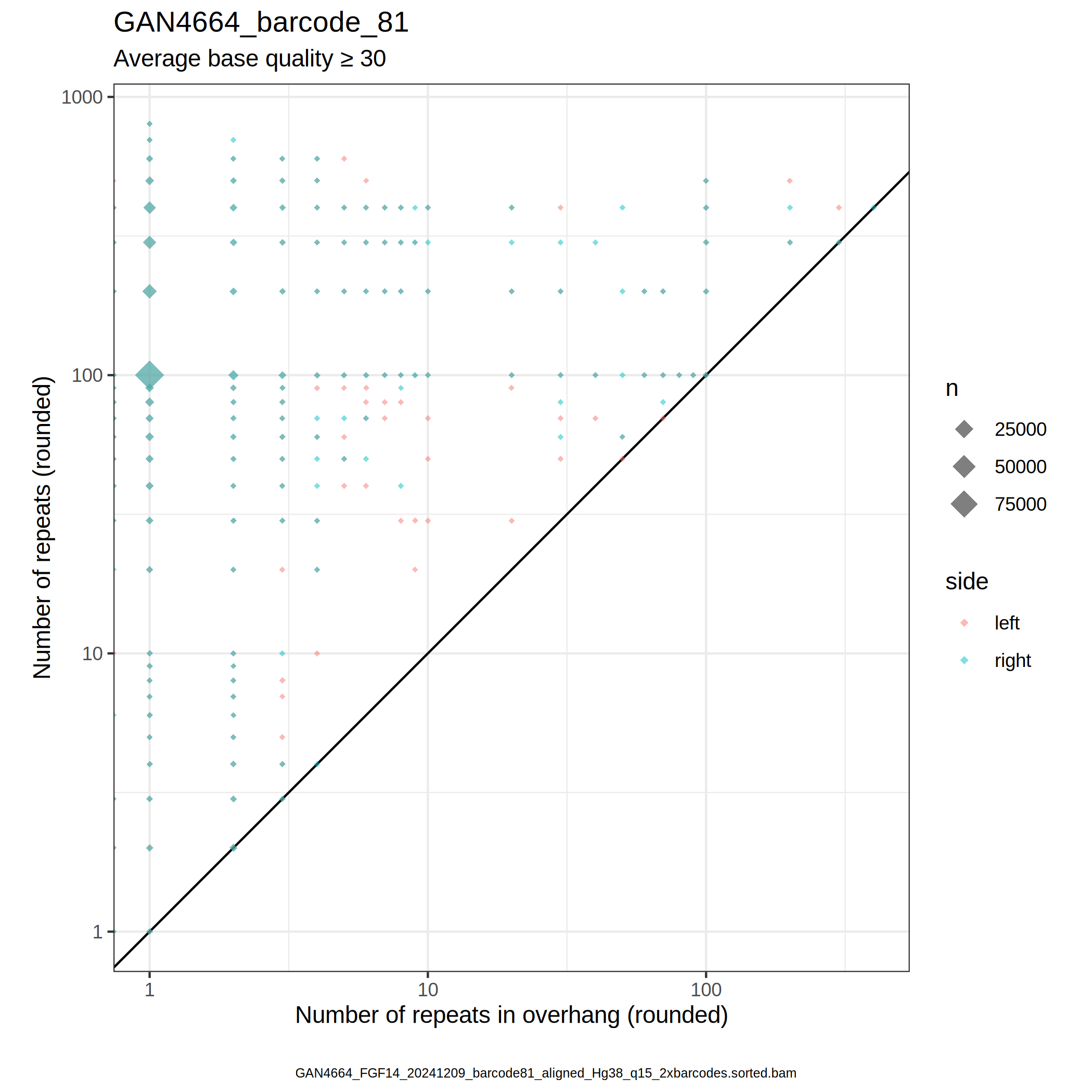


Figure S3: Number of repeats in relation to the number of repeats in the overhang

We also plotted the size of repeats in both strands against each other. Values close to the main diagonal, i.e. scoring high on either strand, indicate potential sporadic matches. In high-quality calls, most cases should be found further away from the main diagonal, especially for longer repeats, see Figure S4.


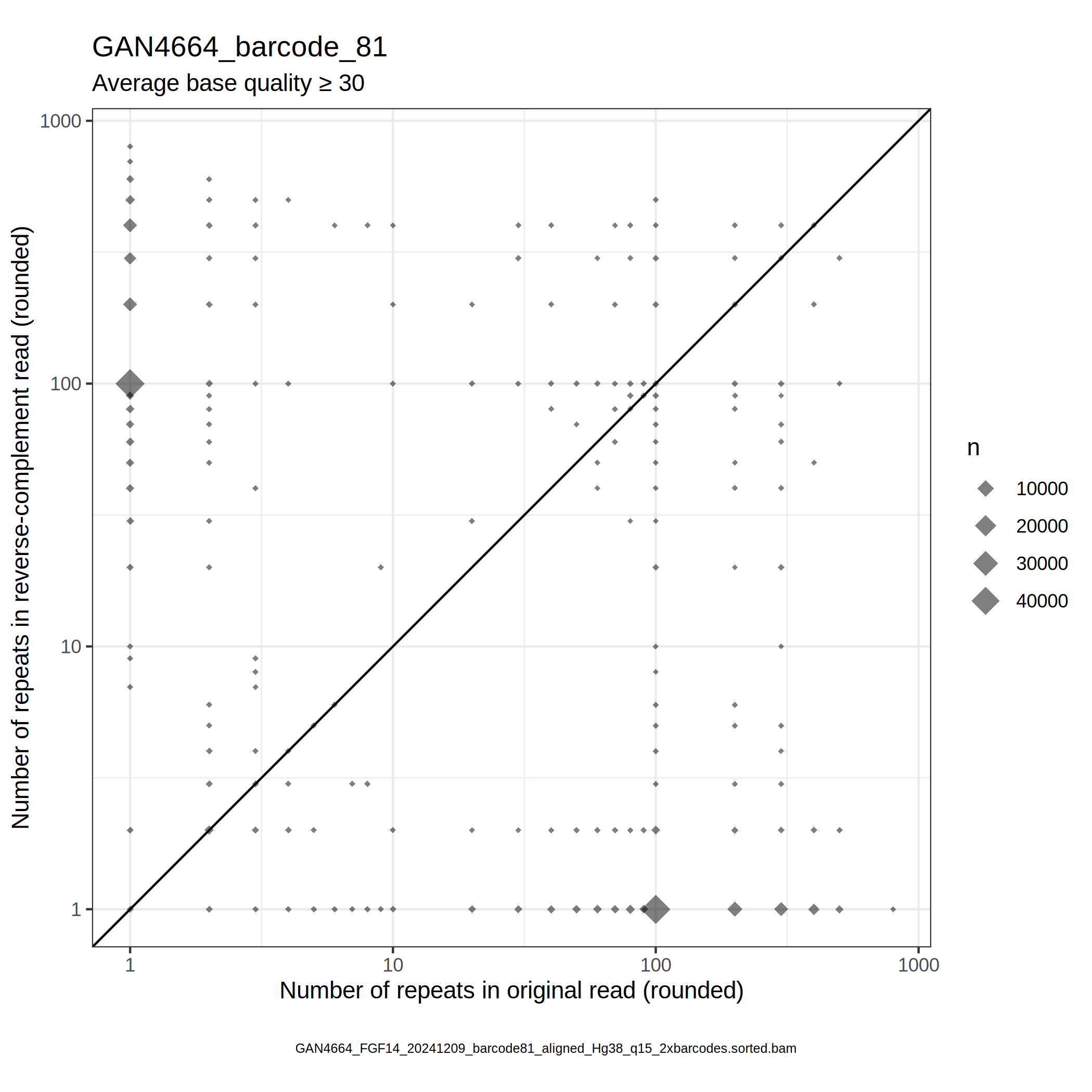


Figure S4: Number of repeats in the reverse-complement reads in relation to the number of repeats in the original read

Assessment

CSW allows for efficient large-scale screening and convenient visual assessment. We observed negligible amounts of repeat regions outside of the calls made by CSW, which were also very short and likely sporadic. We also observed negligible amounts of repeats at read boundaries, so as to be barely visible in the spectrum. Unsurprisingly, the naive peak annotation matched the visually identified peaks very well; sporadic peaks such as 74 in the example above are due to the simplistic peak calling method being used, and are easily discarded visually.

While some of the quality control could be automated within certain parameters, we found visual assessment of the results and quality parameters to be more efficient and insightful, as the results and failure modes can be quite complex, yet easy to comprehend visually.

Discussion of the analytical strategy

Alternative sequencing methods

Bonnet et al. showed that capillary electrophoresis of long-range PCR amplification products underestimated the repeat number whereas Sanger sequencing and Nanopore sequencing resulted in similar size estimates^5^. Even though Bonnet et al. favored Sanger sequencing, Nanopore sequencing has proven advantageous for detecting somatic mosaicism, as it allows for a more precise characterization of repeat length variability at this locus and for confirmation of two alleles being present in case of a homozygous signal obtained with other methods. Furthermore, Nanopore sequencing is becoming increasingly available for clinical applications.

Performance of the CSW algorithm

The CSW algorithm proved highly effective for accurate estimation of repeat length, particularly in overcoming the challenges posed by somatic mosaicism. The simplicity of repeat quantification and peak calling makes this an elegant and fast method for repeat quantification in diagnostic settings. Moreover, this approach enables a visual inspection of the repeat spectrum, which may serve for potential future classification and analysis. However, given known and potential limitations of the adapted algorithm, visual inspection of the repeat sequence in IGV (due to the similarity to the benign repeat motifs and recently described motif interruptions^6^) remains essential for validating results. More advanced calling algorithms might be warranted to appreciate the complexities of the expanded sequences.

GAA-*FGF14* ataxia among sporadic cases stratified by clinical subgroups

In two of the six sporadic cases presenting with at least one typical GAA-*FGF14* ataxia feature (subgroup 1), a pathogenic GAA-*FGF14* expansion was identified. Among sporadic cases with relatively pure cerebellar ataxia but without reported typical features (subgroup 2), the diagnostic yield was 8% (3/38 cases). This subgroup could be clinically challenging, as the combination of absent typical features and a negative family history complicates diagnostic assessment. Furthermore, in 10% (4/38) of this subgroup, an intermediate GAA-*FGF14* expansion of currently uncertain clinical significance was identified. In general, the limited sample size within these subgroups precludes meaningful conclusions. Nevertheless, it is noteworthy that both atypical GAA-*FGF14* cases had a negative family history. Additionally, four of the five total cases with intermediate expansions were sporadic. This renders them particularly challenging cases that require further longitudinal observation and more precise classification in the future.

**
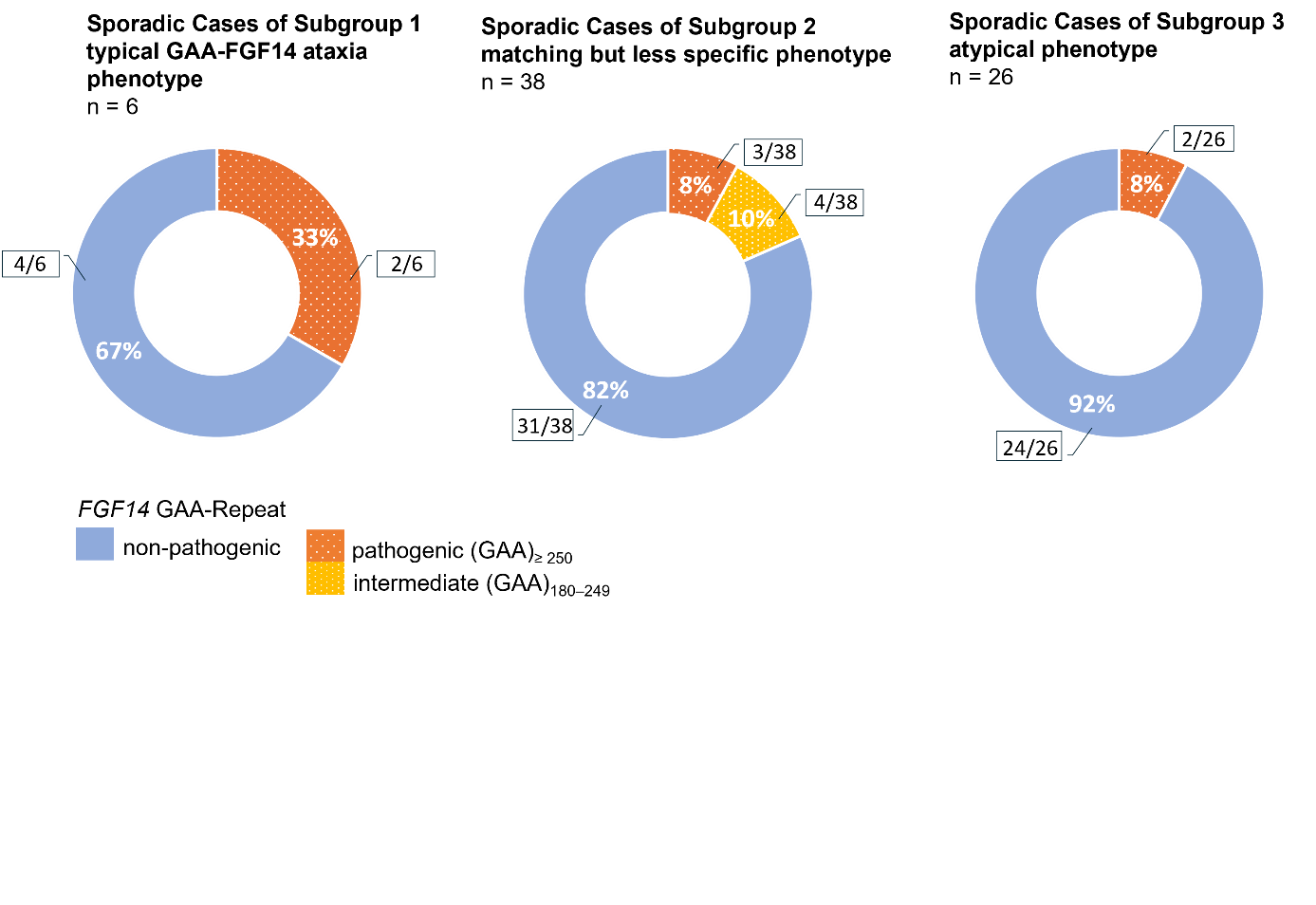
**

Figure S5: GAA-*FGF14* ataxia among sporadic cases stratified by clinical subgroups

Subgroup 1: CA + downbeat nystagmus/episodic component/alcohol sensitivity. Subgroup 2: relatively pure CA with an AAO >30 years. Subgroup 3: CA but rather atypical symptoms for GAA-*FGF14* (e.g. rapid progression, prominent extracerebellar symptoms).

Supplementary Table S1: Overview of prior genetic testing for all patients

Provided in the additional Excel file (Supplementary Table S1.xlsx).

Supplementary Table S2: Examples of exact genotyping using IGV and CSW

| ID | IGV Screenshot^a^ | Screening IGV^b^ | Tandem Repeat Spectrum | Classification |
| --- | --- | --- | --- | --- |
| 02 | 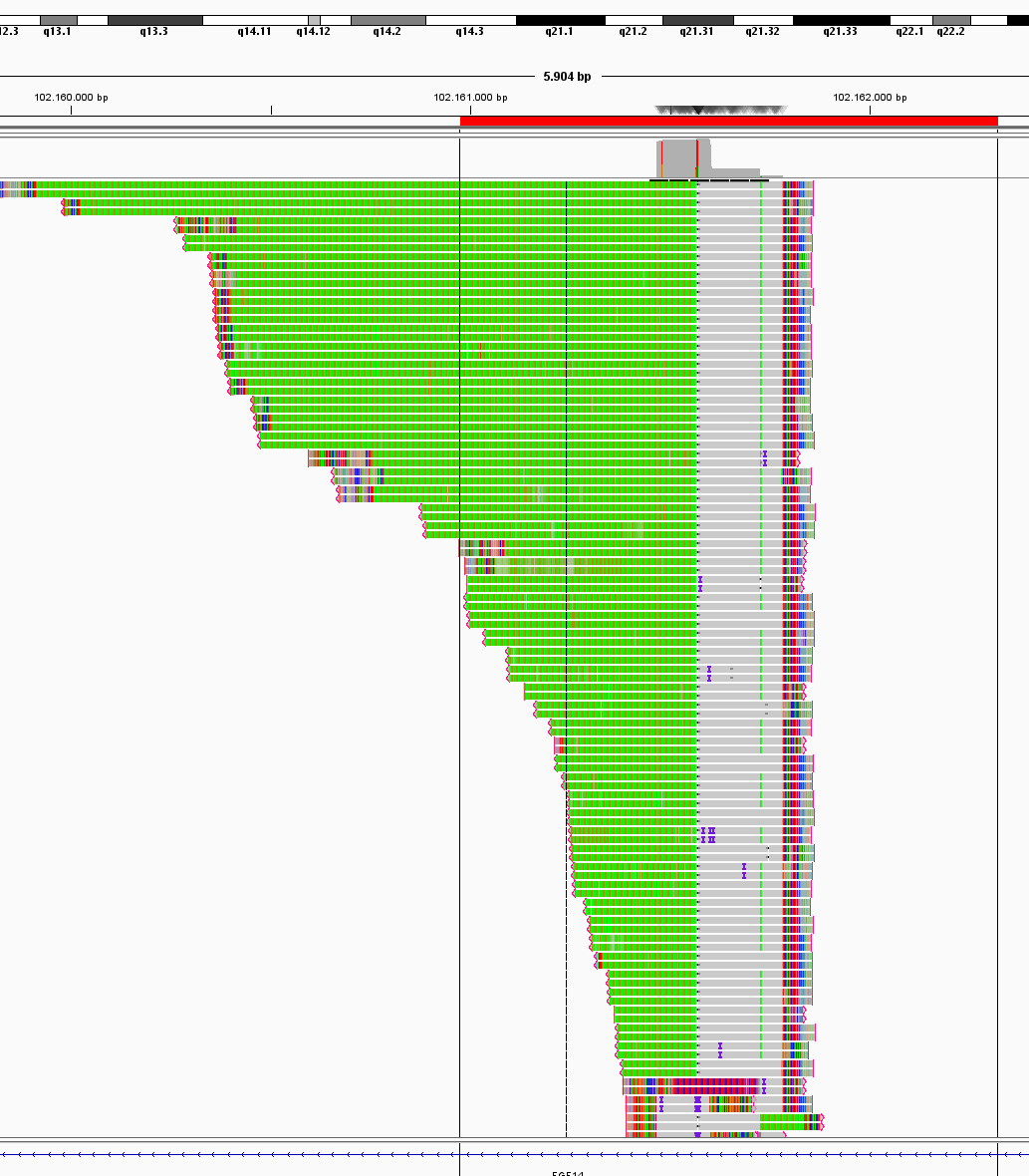 | positive | 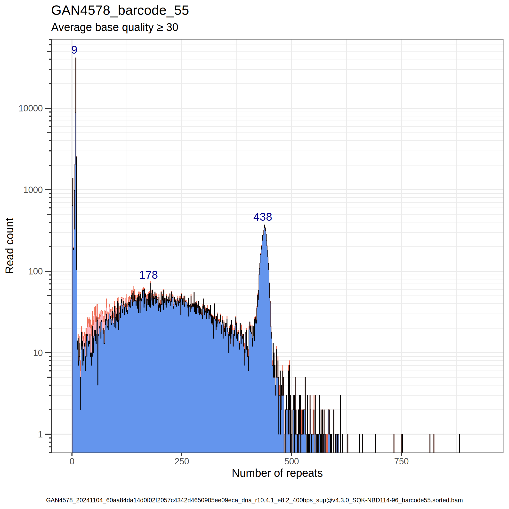 | pathogenic |
| 06 | 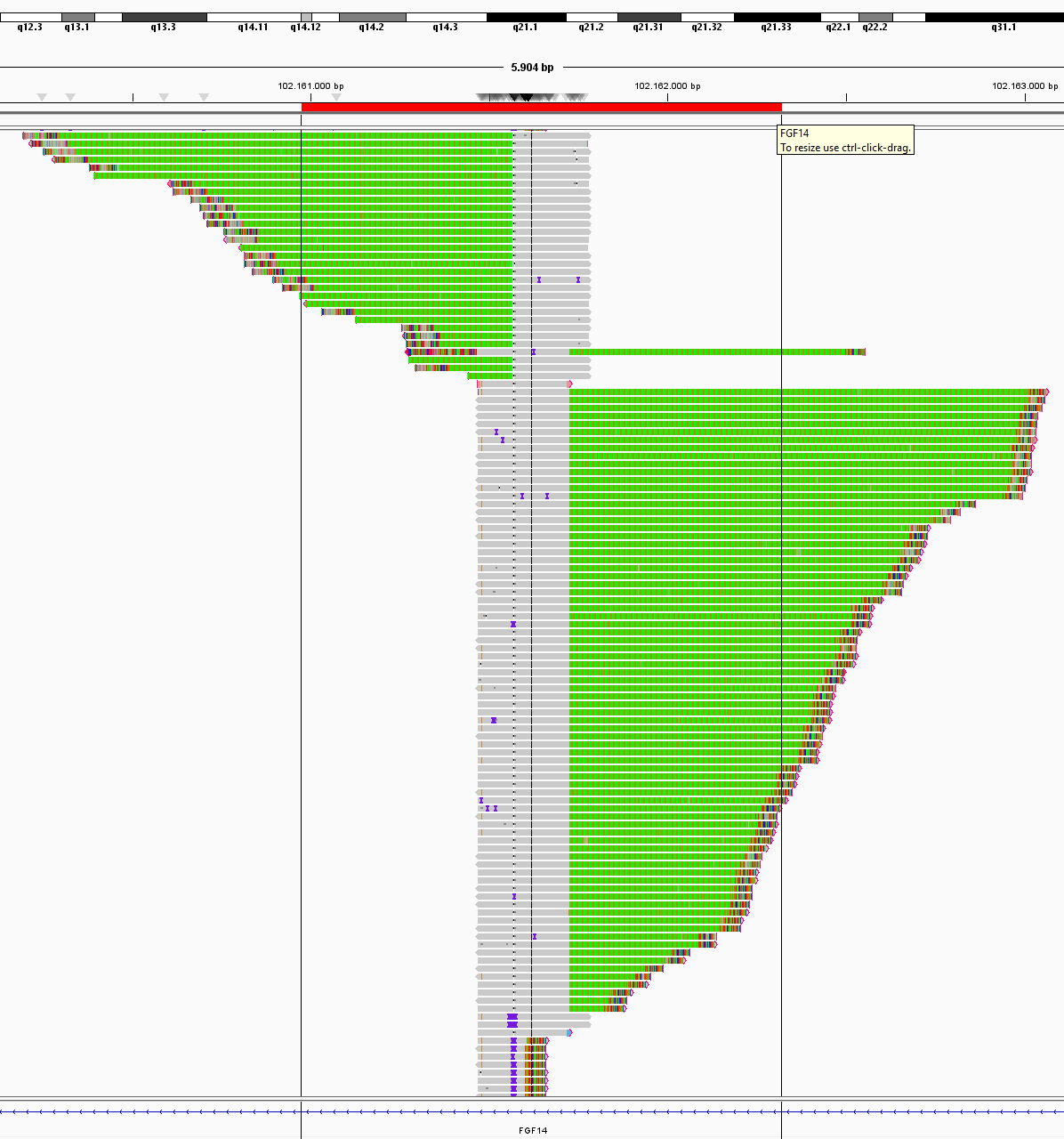 | positive | 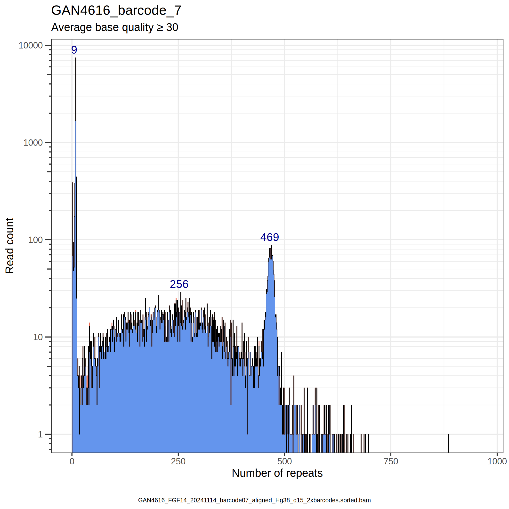 | pathogenic |
| 22 | 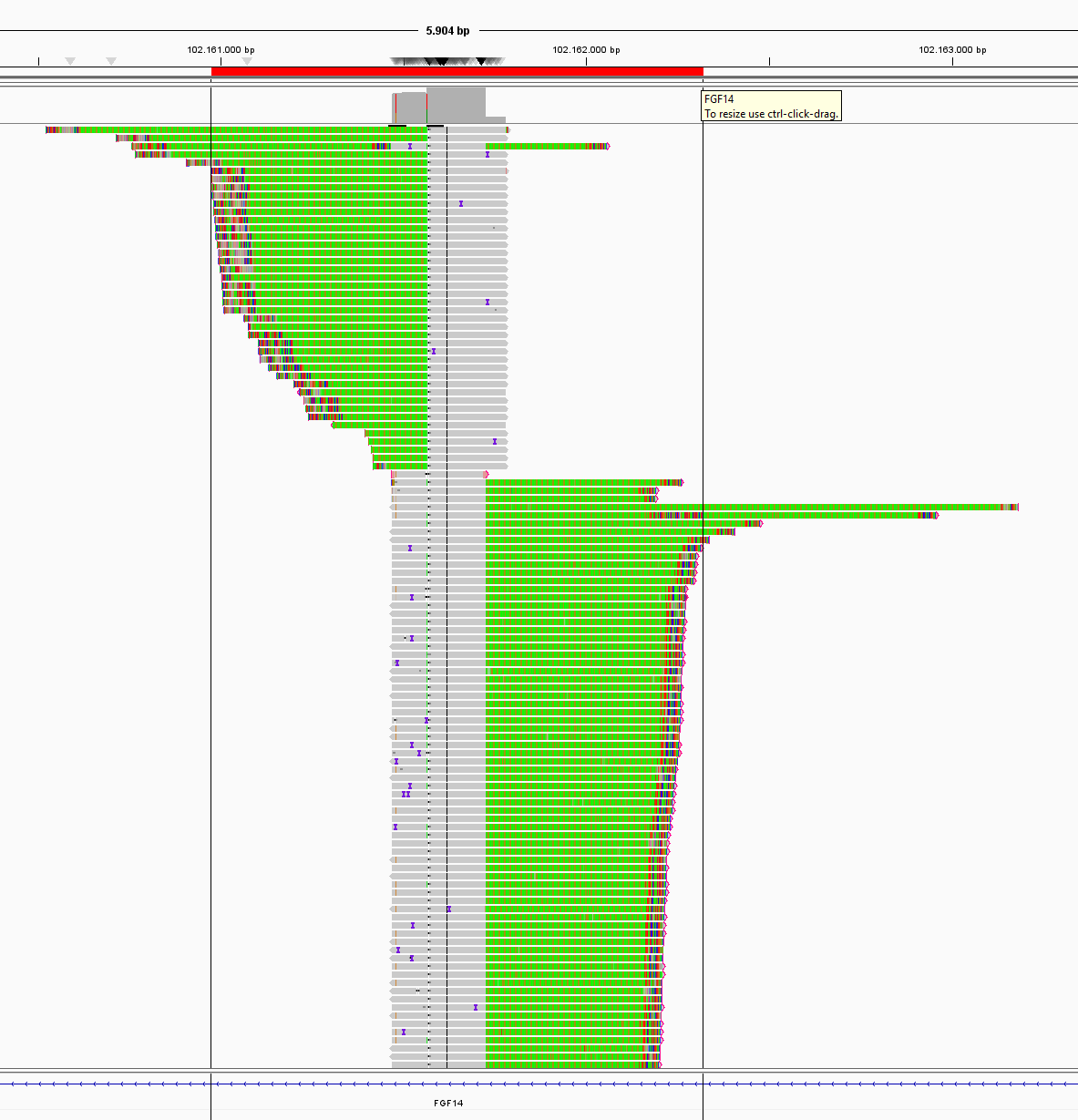 | borderline | 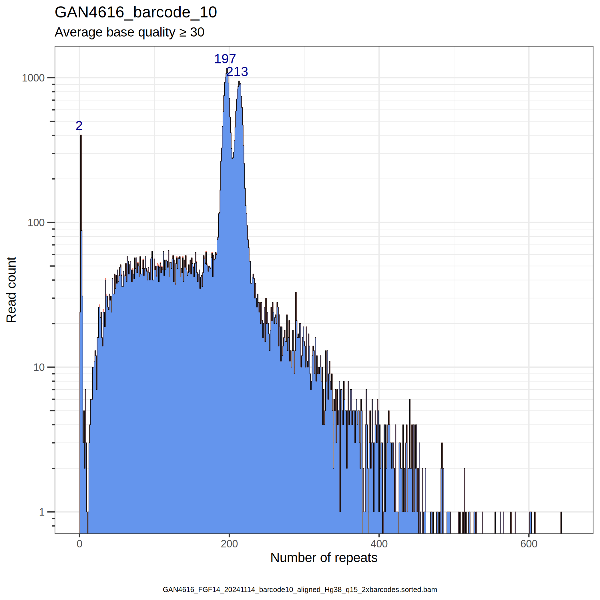 | intermediate |
| 01 | 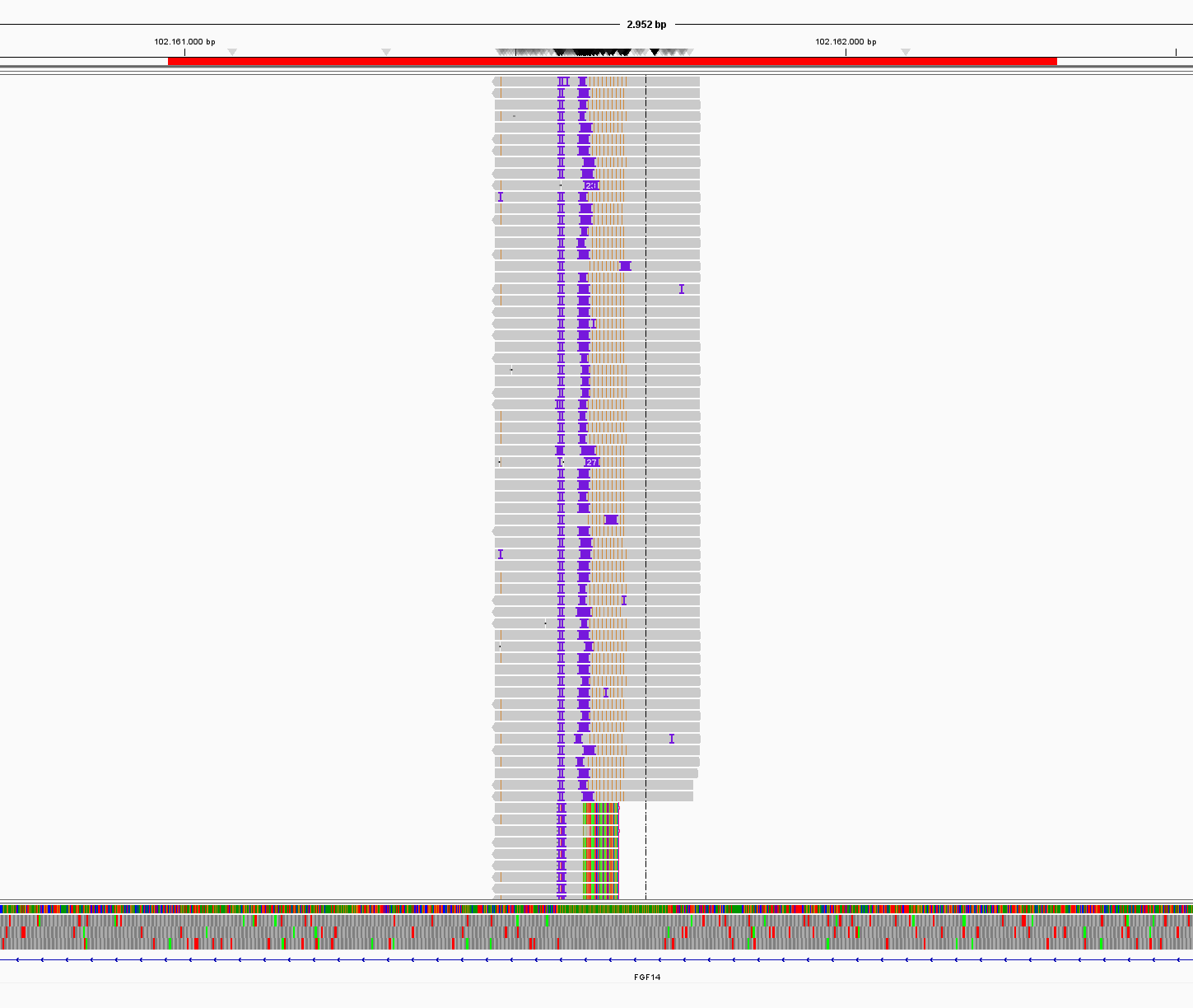 | negative | 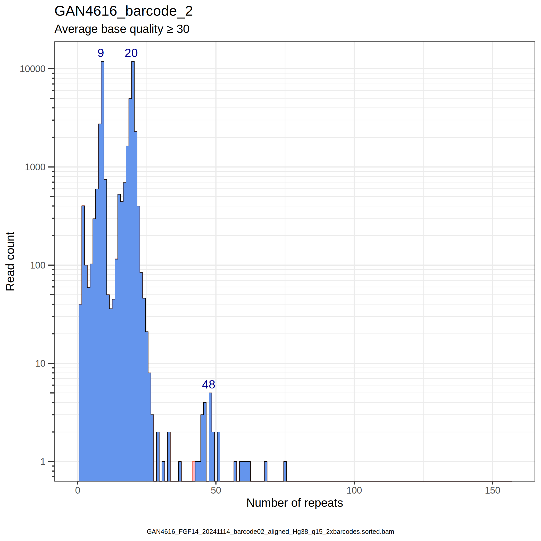 | normal |

^a^ The red horizontal bar demonstrates the range of up to 200 repeats. Cases with repeat lengths beyond this red bar are considered as positive in the screening.

^b^ positive = repeat length >200, negative = repeat length clearly <180, borderline: repeat length around 200

Supplementary Table S3: Clinical and genetic details of the GAA-*FGF14* ataxia cases

| ID | Sex | Symptoms | SARA score^a^ | MRI | AAO | Age at exam | Family  history  for ataxia | Subgroup | Allel 1 approx. GAA  repeat length^b^ | Allel 2 approx. GAA  repeat length^b^ | Zygosity | *FGF14*  Classification^c^ |
| --- | --- | --- | --- | --- | --- | --- | --- | --- | --- | --- | --- | --- |
| 02 | ♂ | slowly progredient CA with intermittent dysarthria, downbeat nystagmus, episodic ataxia, alcohol sensitivity, mild polyneuropathy | 4 | NA | 60 | 76 | father with balance difficulties | 1 | 438 | 9 | heterozygous | pathogenic |
| 03 | ♂ | left-accentuated cerebellar syndrome with downbeat nystagmus, visual disturbance (diplopia, fixation problems), balance disturbance, sensory polyneuropathy | NA | mild  cerebellar atrophy | 64 | 69 | unremarkable | 1 | 311 | 9 | heterozygous | pathogenic |
| 06 | ♀ | initially episodic gait disturbances, recurrent falls, slowly progressive cerebellar syndrome with stance, gait and limb ataxia, mild dysarthria | 9 | no  cerebellar atrophy | 65 | 77 | mother and sister with gait disturbance | 1 | 469 | 9 | heterozygous | pathogenic |
| 09 | ♀ | progressive CA with downbeat nystagmus, stance and gait ataxia | 4 | mild  cerebellar atrophy | 64 | 66 | unremarkable | 1 | 327 | 118 | heterozygous | pathogenic |
| 10 | ♂ | paroxysmal gait and balance disturbances, oculomotor dysfunction and dysarthria (approximately 5–7 times daily, lasting 30 minutes); good response to 4-aminopyridine | NA | unremarkable | 70 | 73 | unknown | 1 | 332 | 20-65, interruption of 14 GAAGAG  sequence  repeats | heterozygous | pathogenic |
| 14 | ♀ | slowly progressive CA with vertigo, gait disturbance, oculomotor dysfunction, dysarthria and polyneuropathy | 11 | unremarkable | 70 | 83 | unremarkable | 2 | 331 | 50 | heterozygous | pathogenic |
| 25 | ♂ | cerebellar syndrome, progressive gait disturbance, vertigo | NA | NA | 60 | 80 | sister with gait disturbance | 2 | 376 | 16 | heterozygous | pathogenic |
| 27 | ♂ | progredient cerebellar ataxia, ataxic gait and prominent balance disturbance, horizontal gaze-evoked nystagmus, polyneuropathy | NA | unremarkable | 40 | 77 | unremarkable | 2 | 362 | 16 | heterozygous | pathogenic |
| 28 | ♂ | cerebellar syndrome, head and voice tremor, gait ataxia, polyneuropathy, microangiopathic leukoencephalopathy | NA | microangiopathiy,  cerebellum unremarkable | 73 | 77 | unremarkable | 2 | 367 | 16-66, interruption of 13 GAAGAG  sequence  repeats | heterozygous | pathogenic |
| 38 | ♂ | CA with progressive stance, gait, and limb ataxia, dysarthria, cerebellar oculomotor dysfunction | 15 | unremarkable | 73 | 81 | mother and two sisters with frequent falls at older age | 2 | 308 | 151 | heterozygous | pathogenic |
| 45 | ♀ | slowly progressive CA with dysarthria, limb and truncal ataxia, inability to walk or stand independently, mild oculomotor dysfunction, vertigo, pyramidal tract signs | >12 | microangiopathy, no notable cerebellar atrophy | 74 | 85 | sister with gait  disorder, AAO 60 | 2 | 287 | 228 | biallelic | pathogenic  reduced penetrance |
| 61 | ♂ | progressive gait and stance ataxia | NA | cerebellar atrophy | 69 | 79 | two  brothers with gait disturbance | 2 | 286 | 18 | heterozygous | pathogenic  reduced penetrance |
| 69 | ♀ | fall tendency, hypokinetic-rigid syndrome, pyramidal tract syndrome, mild cerebellar syndrome, cognitive impairment | NA | MRI consistent with APS, Tau-PET-MRI: tauopathy | 52 | 56 | unremarkable | 3 | 444 | 132 | heterozygous | pathogenic |
| 104 | ♀ | episodic pain, episodic paresis and paresthesia, episodic vertigo, sudden loss of muscle tone and falls, dissociative component | NA | unremarkable | 40 | 56 | unremarkable | 3 | 262 | 9 | heterozygous | pathogenic  reduced penetrance |
| 13 | ♂ | CA with dysarthria, oculomotor dysfunction, gait disturbance, markedly right-sided and arm-predominant hemiataxia | 14,5 | moderate cerebellar atrophy, particulary vermis | 64 | 71 | unremarkable | 2 | 190 | 13-59, interruption of 13 GAAGAG sequence repeats | heterozygous | intermediate |
| 22 | ♀ | CA, dysarthria, diplopia and nystagmus, right-sided dysdiadochokinesia, memory problems | NA | cerebellar degeneration | 48 | 56 | unremarkable | 2 | 197 | 213 | biallelic | intermediate |
| 35^d^ | ♀ | progressive CA with oculomotor dysfunction, dysarthria, limb ataxia, stance and gait ataxia, additional mild extrapyramidal motoric symptoms (bradykinetic movement disorder, rigidity) | 8,5 | cerebellar atrophy and asymmetric cerebellar hyopometabolism (PET/MRI) | 51 | 56 | unremarkable | 2 | 183 | 9 | heterozygous | intermediate |
| 37^e^ | ♂ | slowly progressive CA with gait, balance, and oculomotor dysfunction; subtle pyramidal tract involvement, pallhypesthesia, pronounced calf hypertrophy | 12 | mild cerebellar volume loss | 33 | 44 | brother with slowly progressive gait disorder, AAO 41 | 2 | 186 | 10 | heterozygous | intermediate |
| 57 | ♀ | insidiously progressive cerebellar syndrome with initially increased salivation, unilateral hand tremor and gait disturbance, development of oculomotor dysfunction, dysarthrophonia and dysphagia | NA | cerebellar atrophy | 41 | 44 | unremarkable | 2 | 185 | - | homo  zygous^f^ | intermediate |

CA = cerebellar ataxia, AAO = age at onset, APS= atypical parkinsonian syndrome

^a^ Most recent SARA score, if reported in the neurological examination report; NA indicates that the SARA score was not reported in the available reports

^b^ Finally determined by review of IGV and the Tandem Repeat Spectrum by two independent experienced evaluators (E.K. and D.P.). Local modal values are stated und were used for clinical interpretation. The GAA repeat expansion is, unless otherwise stated, without interruption.

^c^ pathogenic >300 GAA repeats, pathogenic with reduced penetrance 250-299 GAA repeats, intermediate 180-249 GAA repeats

^d^ Additional genetic finding: heterozygous variant of uncertain significance in *ELOVL5*: NM_021814.5: c.140G>A, p.(Trp47*)

^e^ Additional genetic finding: heterozygous intermediate repeat-expansion in *CNBP*, NM_001127192.1:c.-14-806_-14-885CCTG[(68_93)]

^f^ Due to a heterozygous single nucleotide polymorphism (chr13:102161480T>C) it is apparent that two alleles were amplified and sequenced. Hence, this repeat expansion was determined as homozygous.

**References**

1 Mohren L, Erdlenbruch F, Leitão E, et al. Identification and characterisation of pathogenic and non-pathogenic FGF14 repeat expansions. Nat Commun 2024; 15(1):7665. https://doi.org/10.1038/s41467-024-52148-1.

2 Smith TF, Waterman MS. Identification of common molecular subsequences. Journal of Molecular Biology 1981; 147(1):195–97. https://doi.org/10.1016/0022-2836(81)90087-5.

3 Gotoh O. An improved algorithm for matching biological sequences. Journal of Molecular Biology 1982; 162(3):705–08. https://doi.org/10.1016/0022-2836(82)90398-9.

4 Durbin R, Eddy SR, Krogh A, Mitchison G. Biological Sequence Analysis. Cambridge University Press, 2012.

5 Bonnet C, Pellerin D, Roth V, et al. Optimized testing strategy for the diagnosis of GAA-FGF14 ataxia/spinocerebellar ataxia 27B. Sci Rep 2023; 13(1):9737. https://doi.org/10.1038/s41598-023-36654-8.

6 Laß J, Thomsen M, Borsche M, et al. FGF14 repeat length and mosaic interruptions: modifiers of spinocerebellar ataxia 27B? Brain 2025; 148(11):4072–83. https://doi.org/10.1093/brain/awaf183.
